# A little nudge goes a long way: assessing the impact of a microbiology nudge comment on narrow-spectrum antibiotic use in uncomplicated *Streptococcus pneumoniae* bloodstream infections

**DOI:** 10.1017/ash.2025.10137

**Published:** 2025-09-18

**Authors:** Michael O. Akon, Rachel M. Kenney, Nathan A. Everson, Sydney VanDorf, Geehan Suleyman, Robert J. Tibbetts, Anita B. Shallal, Michael P. Veve

**Affiliations:** 1 Department of Pharmacy, Henry Ford Health, Detroit, MI, USA; 2 Department of Infectious Diseases, Henry Ford Health, Detroit, MI, USA; 3 Department of Microbiology, Henry Ford Health, Detroit, MI, USA; 4 Department of Pharmacy Practice, Eugene Applebaum College of Pharmacy and Health Sciences, Wayne State University, Detroit, MI, USA

## Abstract

Narrow-spectrum antibiotic prescribing (ampicillin IV or penicillin IV) was compared before and after implementing an interpretive microbiology comment for uncomplicated *Streptococcus pneumoniae* bloodstream infections. The postintervention group was associated with 4-fold increased odds of de-escalation to narrow-spectrum antibiotics (adjusted odds ratio, 4.66; 95% confidence interval, 1.97–11.00).

## Introduction

*Streptococcus pneumoniae* bloodstream infections (BSI) account for 25–30% of invasive pneumococcal disease and are associated with high mortality, which is concerning among high-risk populations where United States vaccination coverage is estimated at 22.2%.^1,2^ Non-meningitis *S. pneumoniae* BSI are often initially treated with prolonged broad-spectrum antibiotics and represent an opportunity for antimicrobial stewardship intervention.^3,4^

Microbiology nudging is a behavioral intervention strategy that persuades clinicians toward prescribing optimal antibiotic therapy through delibrate microbiology reporting.^5,6^ Literature suggests that nudge comments result in increased optimal antibiotic use and are a practical, low-resource intervention.^7–10^

In 2024, the Henry Ford Health (HFH) antimicrobial stewardship program (ASP) developed and implemented a targeted microbiology comment designed to support optimal antibiotic decision making in *S. pneumoniae* BSI. The study purpose was to evaluate the impact of a targeted microbiology nudge on prescribing behavior.

## Methods

This was an IRB-approved, single pre /posttest quasi-experimental study conducted at a five-hospital health system in Michigan. The HFH ASP primary intervention strategy includes clinical pharmacist audit-and-feedback for sterile site culture results, including the bloodstream.

Hospitalized adult patients ≥18–years with microbiologically confirmed, monomicrobial *S. pneumoniae* BSI were included. The prenudge group time period included 10/01/2022–09/26/2023 while the postnudge was 10/01/2023–10/02/2024. Patients were excluded if they had documented IgE-mediated or severe cutaneous reaction to β-lactam antibiotics, polymicrobial infections, were receiving comfort/hospice care at time of infection, died <48–hours of culture result, or had suspected/confirmed meningitis or endocarditis.

Beginning 9/27/2023, an automated microbiology nudge comment was appended to rapid blood polymerase chain reaction (PCR) results for *S. pneumoniae* in the electronic health record (EHR): “Drugs of choice = Ampicillin IV or Penicillin IV. For meningitis, use max dose Ceftriaxone IV plus Vancomycin IV until susceptibilities known.” Preintervention, microbiology reports stated “Aerobic and anerobic bottles. *Streptococcus pneumoniae*. Susceptibility to follow. 3^rd^ generation cephalosporin IV recommended until susceptibilities available. Add vancomycin IV for patients with meningitis.” The HFH ASP developed a one-page educational handout provided to the pharmacy department through a weekly email for the month before and after nudge implementation. Education was shared with providers on the day of implementation through electronic communication, including pulmonary/critical care leadership, chief medical residents, and infectious disease providers.

The primary outcome was de-escalation to narrow-spectrum within 48–hours of rapid blood PCR results, defined as initiating IV penicillin or IV ampicillin. Patients who received concomitant broad-spectrum antibiotics, defined as ampicillin-sulbactam, cefepime, ceftriaxone, fluoroquinolones, metronidazole, piperacillin/tazobactam, and vancomycin, concomitantly with IV penicillin or IV ampicillin were not considered to meet the de-escalation primary outcome. The BCID2 PCR panel (bioMérieux SA, Marcy-l’Étoile, France) was used for organism identification.

Secondary outcomes included oral switch, or initiation of amoxicillin or penicillin during hospital admission or upon discharge, 30–day all-cause in-hospital mortality, duration of broad-spectrum antibiotics, and length of hospital/intensive care unit (ICU) stay. Treatment-related adverse events included *Clostridioides difficile* infection, severe cutaneous reactions, and hypersensitivity reactions. A treatment duration of ≤10 days was the nonequivalent dependent variable. Dual therapy was defined as receipt of two empiric antibiotics with different mechanisms of action and activity against *S. pneumoniae* for at least 48–hours. Hospice care was defined as receiving palliative care or having a hospice consult placed at the time of infection.

Patient data for screening were acquired using Microsoft SQL Server Management Studio (Microsoft, Redford, WA, USA) based on positive *S. pneumoniae* blood culture results during the study timeframe. Patients that met inclusion criteria had demographic, infection, treatment, and outcome data manually collected from the EHR using a standardized case report form.

Descriptive statistics (proportions [%], median [IQR]) were used to describe the patient population; bivariate comparisons were performed using the Mann–Whitney *U* test, or Pearson *χ*^2^ test, or Fisher’s exact test. To identify factors independently associated with de-escalation, variables with a *P*–value of <0.2 in bivariate analyses were considered for inclusion in a multivariable logistic regression model. A *P*–value of <0.05 was considered statistically significant for all analyses. Statistical tests were conducted using SPSS Statistics v. 30.0 (IBM Corp., Armonk, NY, USA).

## Results

There were 105 patients included: 54 (51.4%) patients in the prenudge group and 51 (48.6%) in the postnudge group. Baseline characteristics of the pre- and postnudge groups are in Table 1. Of the total study population, 101 (96.2%) were diagnosed with suspected/confirmed pneumonia, the median (IQR) age was 63 (25–95) years, 51 (48.5%) were women, and 43 (40.9%) required ICU admission at time of infection. Of the patients admitted to the ICU, 9 (20.9%) required mechanical ventilation, and 17 (39.5%) received vasopressors. There was a lower proportion of patients who received dual therapy in the prenudge group when compared to the postnudge group (50.0% vs 74.5%; *P* = 0.010). There was a higher proportion of patients in the prenudge group who received corticosteroids compared to the postnudge group (59.3% vs 47.1%, *P* = 0.210).

**Table 1.** Baseline characteristics and outcomes of patients before and after implementation of a *Streptococcus pneumoniae* blood PCR microbiology nudge

Variable (*n*, % or median, IQR)	Prenudge (*n* = 54)	Postnudge (*n* = 51)	*P*-value
Age, years	62 (49.50–71.50)	63 (56–70)	0.665
Sex, woman	26 (48.1%)	25 (49.0%)	0.929
Charlson Comorbidity Index	4 (2–7)	4 (3–7)	0.218
Corticosteroid use	32 (59.3%)	24 (47.1%)	0.210
Received dual antibiotic therapy	27 (50%)	38 (74.5%)	0.010
Required mechanical ventilation	5 (9.3%)	4 (7.8%)	0.796
Required vasopressor therapy	11 (20.4%)	6 (11.8%)	0.232
ID consultation	36 (66.7%)	30 (58.8%)	0.406
Received *S. pneumoniae* vaccination	4 (7.4%)	6 (11.8%)	0.447
Admitted to an ICU	23 (42.6%)	20 (39.2%)	0.725
Total treatment ≤10 d^†^	20 (37%)	24 (47.1%)	0.298
Narrow-spectrum antibiotic use Ampicillin IV Penicillin IV	16 (29.6%) 12 (22.2%) 4 (7.4%)	34 (66.7%) 34 (100%) 0	<0.001 – –
*Clostridioides difficile* infection Severe cutaneous reaction Hypersensitivity reaction	1 (2%) 0 0	1 (2%) 0 0	1.0 – –

**Abbreviations:** PCR, polymerase chain reaction; ICU, intensive care unit; ID, infectious diseases; ADE, adverse drug events

†Total treatment ≤10 days was selected as the non-equivalent dependent variable and was not different between groups.

The primary outcome of de-escalation within 48–hrs to ampicillin or penicillin therapy was observed in 16 (29.6%) pre vs 34 (66.7%) postnudge patients (*P* < 0.001). Oral switch, a secondary outcome, was observed in 19 (35.2%) pre vs 29 (56.9%) post-nudge patients (*P* = 0.026). The median (IQR) broad-spectrum antibiotic duration (days) was 8 (4–12.25) pre vs 3 (1–9) post-nudge patients (*P* < 0.001). Median (IQR) hospital LOS (8 [5–16] days vs 6 [4–10] days, *P* = 0.083), ICU LOS (7 [3–15] vs 4 [3–14], *P* = 0.292), all-cause mortality (1.9% vs 7.8%, *P* = 0.150), and all-cause readmission (16.7% vs 7.8%, *P* = 0.170) were not different between the pre- and postnudge groups, respectively. After adjusting for no steroid use and vasopressor use, the nudge comment was associated with a 4.6-fold (95%CI, 1.97–11.00) increased odds of de-escalation to narrow-spectrum antibiotics (Table 2).

**Table 2.** Variables associated with 48–hour de-escalation to narrow spectrum for *S. pneumoniae* bacteremia

Variable	unAdjOR (95%CI)	*P*-value	adjOR (95%CI)
Postnudge group	4.75 (2.08–10.84)	<0.001	4.66 (1.97–11.00)
No corticosteroid use^†^	3.36 (1.50–7.46)	0.003	3.30 (1.38–7.75)
Required vasopressor therapy^†^	0.19 (0.05–0.697)	0.007	Not Tested
Received dual antibiotic therapy	1.65 (0.74–3.66)	0.220	Not Tested

Hosmer-Lemeshow Goodness of Fit test results: *χ*^2^, 0.051; *P* = 0.975.

†The variables “no corticosteroid use” and “required vasopressor therapy” were identified to be colinear. As such, “no corticosteroid use” was included into the model due to a greater magnitude of effect.

## Discussion

This study demonstrated that a *S. pneumoniae* BSI microbiology nudge was associated with 4.6-fold increased odds of de-escalation to narrow-spectrum antibiotics, with no difference in clinical outcomes. The population evaluated primarily included critically ill patients who required either mechanical ventilation and/or vasopressor support. There was a higher proportion of patients in the postnudge patients who received dual therapy; however, the microbiology nudge was independently associated with de-escalation in multivariable regression analysis. Patients with a nudge comment had a clinically significant but not statistically significant reduction in hospital and ICU LOS. This suggests that nudging can facilitate medical decision-making and improve efficiency to get patients on optimal therapy more promptly. Importantly, patients who are de-escalated to IV penicillin or IV ampicillin can require additional infusions per day that may be associated with additional product waste or patient sleep interruption. The findings of this study reaffirm that microbiology nudges are simple and highly effective tools to improve antibiotic prescribing with no negative impact on patient outcomes.^5–10^

This study has several limitations. The HFH ASP has a history of leveraging microbiology nudges, including appending comments to PCR results, that may limit external validity. These findings are subject to maturation and regression to the mean given the design. The design is also subject to potential biases in data collection and quality; however, a single data abstractor was used with standardized definitions. While these results suggest a *S. pneumoniae* BSI nudge influenced prescribing, more robust data are warranted given small study size.

## Conclusion

This study adds to growing literature demonstrating the effectiveness of targeted microbiology interventions in guiding prescribing practices for various clinical syndromes.
